# Elevated heparanase expression is associated with poor prognosis in breast cancer: a study based on systematic review and TCGA data

**DOI:** 10.18632/oncotarget.16575

**Published:** 2017-03-25

**Authors:** Xu Sun, Ganlin Zhang, Jiayun Nian, Mingwei Yu, Shijian Chen, Yi Zhang, Guowang Yang, Lin Yang, Peiyu Cheng, Chen Yan, Yunfei Ma, Hui Meng, Xiaomin Wang, Jin-Ping Li

**Affiliations:** ^1^ Department of Oncology, Beijing Hospital of Traditional Chinese Medicine Affiliated to Capital Medical University, Beijing, China; ^2^ School of Graduates, Beijing University of Chinese Medicine, Beijing, China; ^3^ Department of Neurology, Fourth Affiliated Hospital of Guangxi Medical University, Liuzhou, China; ^4^ Department of Medical Biochemistry and Microbiology, Uppsala University, Uppsala, Sweden

**Keywords:** heparanase, breast cancer, prognosis

## Abstract

Heparanase promotes tumorigenesis, angiogenesis, and metastasis. Here, we conducted a study based on systematic review and the Cancer Genome Atlas (TCGA) data that examined heparanase expression in clinical samples to determine its prognostic value. According to the meta-analysis and TCGA data, we found that heparanase expression was up-regulated in most breast cancer specimens, and elevated heparanase expression was associated with increased lymph node metastasis, larger tumor size, higher histological grade, and poor survival. These results suggest that targeting heparanase might improve treatments for breast cancer patients.

## INTRODUCTION

Despite significant progress in the diagnosis and treatment of breast cancer in recent years, it remains the leading cause of cancer-related death in women worldwide [1]. Invasion and metastasis play key roles in malignant tumor pathology and are difficult to treat in clinical practice. The identification of new molecular targets with high prognostic values, particularly targets related to invasion and metastasis, wound help to improve breast cancer treatment.

Heparan sulfate (HS) is an important proteoglycan in the basal membrane and extracellular matrix (ECM), and its roles in building the cellular microenvironment and in cell signaling have been characterized by a number of studies [2–4]. HS acts as a cytokine repository, binding to basic fibroblast growth factor (bFGF), vascular endothelial growth factor (VEGF), keratinocyte growth factor (KGF), and hepatocyte growth factor (HGF) [5–7]. Furthermore, the activity of heparanase (HPSE), the only endoglucuronidase that specifically cleaves HS, is closely related to growth and metastasis in tumor cells [8–10]. Large preclinical studies have shown that HPSE can promote tumor cell metastasis by degrading the ECM, which leads to the activation of HS-bound cytokines and boosts cell proliferation and tumor angiogenesis [11–13]. Some evidence also suggests that high HPSE expression is correlated with increases in tumor cell metastasis and poor prognosis [10, 14].

The relationships between HPSE and cell growth and metastasis have been characterized for a variety of tumor types [15], and its role in breast cancer progression has recently received increasing attention [10, 16–18]. It is currently thought that estrogen receptor (ER) status in breast cancer is related to HPSE expression [18, 19]. In addition, experiments using a variety of tumor cells and related animal models have found that high HPSE expression is associated with increases tumor cell metastasis and chemo-resistance [20, 21]. However, clinical evidence is limited, and reports regarding the effects of HPSE are not always consistent [22, 23]. Therefore, in this study, we analyzed studies and TCGA data of HPSE expression in breast cancer to evaluate its prognostic value.

## RESULTS

### HPSE and breast cancer: a systematic review and meta-analysis

#### Study selection

**Figure 1:**
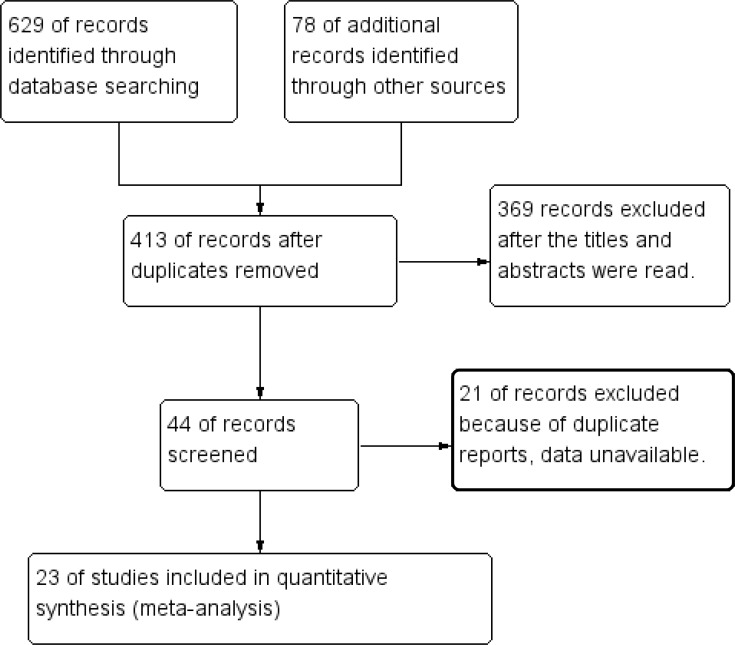
Flow diagram of the study selection process

A total of 629 articles were initially retrieved from database searches, and 78 additional articles were retrieved manually. After duplicate articles were excluded, 23 of the remaining 413 articles, 13 and 10 of which were obtained from English and Chinese databases, respectively, were selected for meta-analysis [10, 16–18, 22–40]. A diagram of the study selection process is shown in Figure 1.

### Characteristics of the included studies

In total, 2,905 subjects from 7 countries and 23 research centers were enrolled in this systematic review. Eighteen studies used immunohistochemical (IHC) staining to detect HPSE protein expression in different tissues, 4 studies used reverse transcription-polymerase chain reaction (RT-PCR) to evaluate HPSE mRNA expression in tissues, and 1 study examined HPSE expression in blood samples by RT-PCR. We analyzed data as originally reported for the high or positive HPSE expression groups in these studies. The characteristics of the included studies are shown in Table 1.

**Table 1:** Characteristics of included studies

NO	Author	Country	Research center	Study period	Cases	Specimen method	Expression difference	Histology grade^c^ (1,2/3)	LNM^a^	Tumor size^b^ (cm) cut-off	ER^a^	HER-2^a^	5-year OS (dead/alive)^d^	NOS
1	Vlodavsky I 1999 [10]	Israel	Hadassah-Hebrew University Hospital	NA	9	tissue PCR	BC VS NT	NA	NA	NA	NA	NA	NA	4
2	Maxhimer J 2002[16]	USA	Rush Presbyterian St Luke's Medical Center	NA	67	tissue IHC	BC VS NT BC VS BBT	NA	P(16/30) N(5/21)	L(18/30) S(3/21) 1 cm	NA	NA	NA	4
3	Zhang Y 2003[24]	China	Changhai Hospital & Changzheng Hospital	1995-2001	108	tissue IHC	BC VS BCAT	H(13/15) L(19/39)	P(24/33) N(8/21)	L(25/35) S(7/19) 2 cm	P(15/29) N(17/25)	NA	H(13:19) L(3:19)	8
4	Liu Z 2004[25]	China	Henan Provincial Tumor Hospital	1993-1997	120	tissue IHC	BC VS BCAT	H(37/37) L(41/83)	P(65/78) N(13/42)	L(69/93) S(9/27) 2 cm	NA	NA	H(34:44) L(3:39)	5
5	Maxhimer J 2005[17]	USA	Rush University Medical Center	NA	57	tissue IHC	BC VS NT	NA	NA	NA	NA	NA	NA	5
6	Zhao J 2006[27]	China	The Third Affiliated Hospital of Fujian Medical University	1998-2003	90	tissue PCR	NA	H(20/21) L(33/59)	P(32/39) N(21/41)	L(41/54) S(12/26) 2 cm	NA	NA	NA	4
7	Imada T 2006[26]	Japan	Okayama University Hospital	2000-2002	103	tissue IHC	NA	NA	P(15/26) N(17/71)	L(14/32) S(15/27) 2 cm	P(27/73) N(8/30)	P(17/40) N(16/53)	NA	8
8	Theodoro T 2007[29]	Brazil	Faculdade de Medicina do ABC	NA	50	serum PCR	BC VS NT	NA	NA	NA	NA	NA	NA	4
9	Li Y 2007[28]	China	Liaohua Hospital	1995-2006	116	tissue IHC	BC VS BBT	NA	P(51/59) N(13/37)	L(39/46) S(25/50) 3 cm	NA	NA	NA	6
10	Davidson B 2007[23]	Norway	National Hospital-Norwegian Radium Hospital	1998-2002	41	tissue IHC	NA	NA	NA	NA	NA	NA	H(78 m^e^) L(116 m)	6
11	Cohen I 2007[18]	Israel	Hadassah Medical Center	NA	214	tissue IHC	BC VS NT	NA	NA	NA	P(59/136) N(12/78)	NA	NA	6
12	Zheng X 2008[31]	China	The Affiliated Hospital of Guizhou Medical University	2004-2005	81	tissue IHC	BC VS BBT	NA	NA	NA	NA	NA	NA	6
13	Wang H 2008[30]	China	The Fourth Hospital of Hebei Medical University	2007-2007	62	tissue PCR	BC VS BCAT(> 5cm)	H(5/6) L(14/25)	P(15/18) N(4/13)	L(18/24) S(1/7) 2 cm	P(15/24) N(4/7)	P(17/25) N(2/6)		8
14	Huan D 2010[33]	China	Fengtian Affiliated Hospital of Shenyang Medical College	2002-2007	110	tissue IHC	BC VS BCAT(>5cm)	H(10/12) L(34/49)	P(26/34) N(29/46)	L(42/59) S(13/21) 2 cm	NA	NA	NA	5
15	Chen L 2010[32]	China	Liaoning Tumor Hospital	1995-2009	95	tissue IHC	BC VS BCAT(>5cm)	H(29/29) L(35/66)	P(46/50) N(18/45)	L(27/30) S(37/65) 3 cm	NA	NA	H(22:42) L(3:28)	5
16	Wang H 2012[34]	China	The First Hospital of China Medical University	2007-2009	124	tissue IHC	BC VS BCAT(>5cm)	H(23/26) L(43/68)	P(48/56) N(15/38)	L(27/34) S(39/60) 3 cm	NA	NA	NA	7
17	Wang X 2012[35]	China	Zhumadian First People's Hospital	2010-2010	180	tissue IHC	BC VS BCAT (<2cm,>5cm)	H(17/17) L(25/43)	P(28/32) N(13/28)	L(33/39) S(8/21) 2 cm	NA	NA	NA	8
18	Tang D 2013[22]	China	The Third Affiliated Hospital of Harbin Medical University	2004-2006	239	tissue IHC	BC VS BCAT	NA	P(34/46) N(32/59)	L(51/71) S(15/34) 2 cm	P(39/63) N(27/42)	P(20/30) N(46/75)	H(18:48) L(6:33)	7
19	Tang D 2014[37]	China	Tumor Hospital of Harbin Medical University	2011-2012	225	tissue IHC	NA	H(49/65) L(52/91)	P(64/86) N(37/70)	L(64/89) S(37/67) 2 cm	P(35/60) N(66/96)	P(31/42) N(70/114)	NA	7
20	Gawthorpe S 2014[36]	UK	Russell's Hall Hospital	2000-2004	236	tissue IHC	NA	NA	NA	L(18/33) S(21/71) 2 cm	NA	NA	NA	7
21	Zhang P 2015[38]	China	The Third Affiliated Hospital of Xinxiang Medical University	2013-2013	100	tissue PCR	BC VS BCAT	NA	P(27/32) N(8/18)	NA	NA	NA	NA	8
22	Yue X 2016[40]	China	First Affiliated Hospital of Bengbu Medical College	2006-2010	400	tissue IHC	BC VS BCAT	H(54/61) L(74/139)	P(83/104) N(48/96)	L(88/109) S(43/91) 2 cm	NA	NA	NA	8
23	Song H 2016[39]	China	The First Affiliated Hospital of Henan University	2012-2014	78	tissue IHC	NA	NA	P(29/36) N(25/42)	L(42/56) S(12/22) 2 cm	P(41/55) N(13/23)	P(47/58) N(7/20)	NA	6

^a^*P*: Positive; N: Negative

^b^L: Large; S: Small

^c^H: High grade (3); L: Low grade (1,2)

^d^H: High expression; L: Low expression

^e^m: Month.

PCR: Polymerase chain reaction; IHC: Immunohistochemistry; NA: Not available; BC: Breast cancer; NT: Normal tissue; BBT: Benign breast tumor; BCAT: Breast cancer adjacent tissue; NOS: Newcastle-Ottawa Scale

### Heparanase is up-regulated in breast cancer specimens

**Figure 2:**
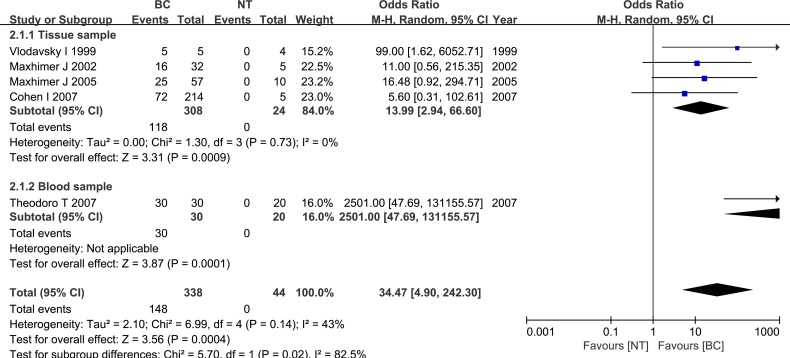
Meta-analysis of HPSE expression in breast cancer and normal breast tissue

Five studies examined differences in HPSE expression between breast cancer specimens and normal breast specimens. These studies included 338 breast cancer tissue specimens and 44 normal breast tissue specimens. HPSE expression was elevated in breast cancer specimens compared to normal specimens (OR = 34.47, 95% CI = 4.90 – 242.30, *P* = 0.0004), and the inter-study heterogeneity was relatively small (*P* = 0.14). Subgroup analysis based on sample type indicated that heparanase was up-regulated in both tissue and blood samples. These results are shown in Figure 2.

**Figure 3:**
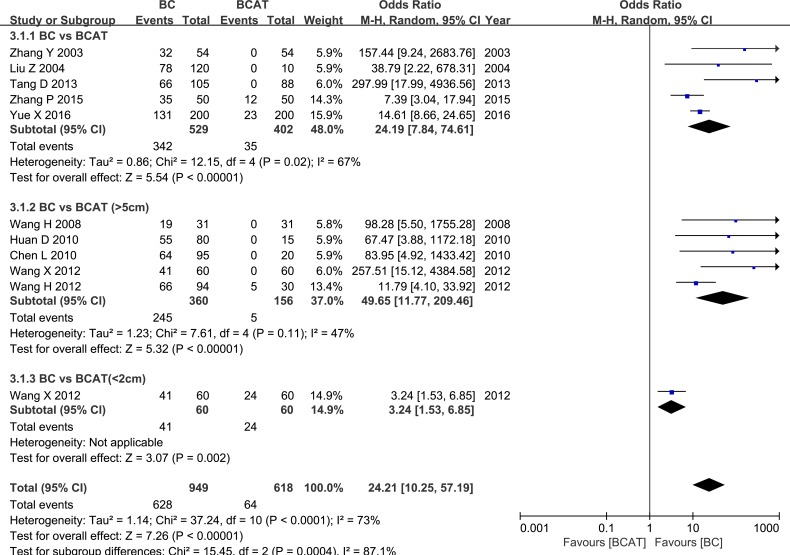
Meta-analysis of HPSE expression in breast cancer and adjacent tissues

**Figure 4:**
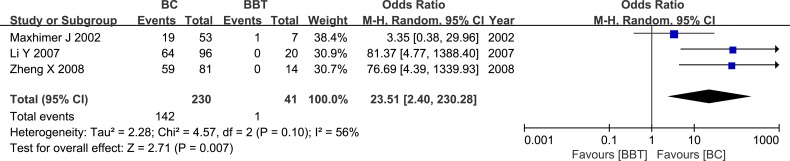
Meta-analysis of HPSE expression in breast cancer and benign breast tumor tissue

Five studies examined HPSE expression in breast cancer (BC) and breast cancer-adjacent normal (BCAT) tissues. Another five studies evaluated BCAT located more than 5 cm from the cancer, and 1 of the studies also evaluated BCAT less than 2 cm from the cancer. Meta-analysis revealed that HPSE expression was higher in BC tissue than in BCAT (OR = 24.19, 95% CI = 7.84 – 74.61, *P* < 0.00001; OR = 49.65, 95% CI = 11.77 – 209.46, *P <* 0.00001; OR = 3.24, 95% CI = 1.53 – 6.85, *P* = 0.002). These results are shown in Figure 3. HPSE expression was similarly elevated in breast cancer tissue compared to benign breast tumor tissue (OR = 23.51, 95% CI = 2.40 – 230.28, *P* = 0.007, Figure 4). These results strongly suggest that HPSE is up-regulated in breast cancer specimens (Table 2).

**Table 2:** Meta-analysis results

Categories	Outcome	No. of Studies/patients	OR (95% CI)	*P*-Value	Heterogeneity	
					*I*^2^	*P*-Value
HPSE is up-regulated in breast cancer specimens	BC vs NT	5/382	34.47 (4.90-242.30)	0.0004	43%	0.14
	BC vs BCAT	10/1567	24.21 (10.25-57.19)	<0.00001	73%	< 0.0001
	BC vs BBT	3/271	23.51 (2.40-230.28)	0.007	56%	0.10
HPSE expression is associated with clinicopathological features of breast cancer	Histological grade (1 vs 2/3)	10/951	6.22 (3.15-12.27)	<0.00001	47%	0.05
	Lymph node metastasis (positive vs negative)	16/1447	4.97 (3.59-6.87)	<0.00001	41%	0.04
	Tumor size (smaller vs larger)	16/1465	3.35 (2.39-4.68)	<0.00001	45%	0.03
	ER (positive vs negative)	7/741	1.55 (0.91-2.63)	0.11	57%	0.03
	Her-2 (positive vs negative)	5/463	2.29 (1.23-4.27)	0.009	47%	0.11
HPSE is correlated with poor 5-year survival	5-year survival (HPSE(+) vs HPSE(−))	4/374	0.23 (0.12-0.47)	<0.00001	21%	0.28

BC: Breast cancer; NT: Normal tissue; BBT: Benign breast tumor; BCAT: Breast cancer adjacent tissue; ER: Estrogen receptor status

### Heparanase expression is associated with clinicopathological features of breast cancer

**Figure 5:**
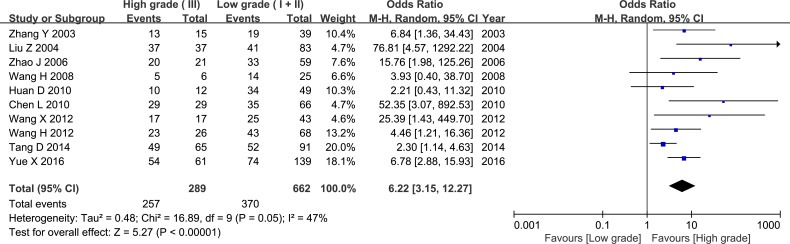
Meta-analysis of HPSE expression and histology grade

**Figure 6:**
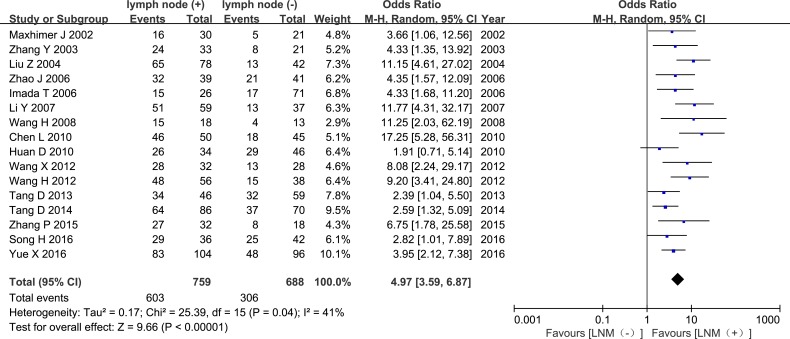
Meta-analysis of HPSE expression in tissues from breast cancer patients with or without lymph node metastasis

**Figure 7:**
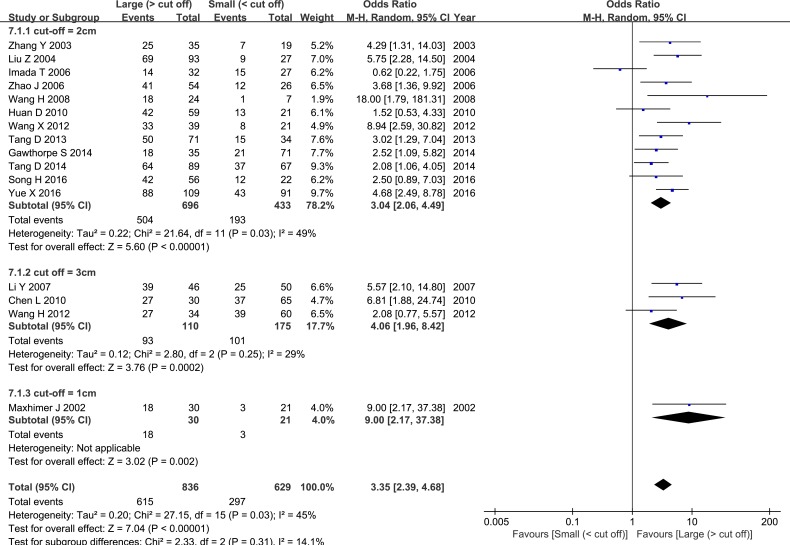
Meta-analysis of tumor size and HPSE expression

The pooled OR revealed that elevated HPSE expression was associated with higher histological grade and increased lymph node metastasis (LNM) (OR = 6.22, 95% CI = 3.15 – 12.27, *P* < 0.00001, Figure 5; OR = 4.97, 95% CI = 3.59 – 6.87, *P* < 0.00001, Figure 6). Sixteen studies evaluated associations between tumor size and HPSE expression. Subgroup analysis using the predetermined cut-off value revealed that high HPSE expression was associated with larger tumor sizes without significant heterogeneity (OR = 3.35, 95% CI = 2.39 – 4.68, *P* < 0.00001, I^2^ = 45%, Figure 7). Together, these findings suggest that high HPSE expression is associated with more aggressive biological characteristics in breast cancer (Table 2).

**Figure 8:**
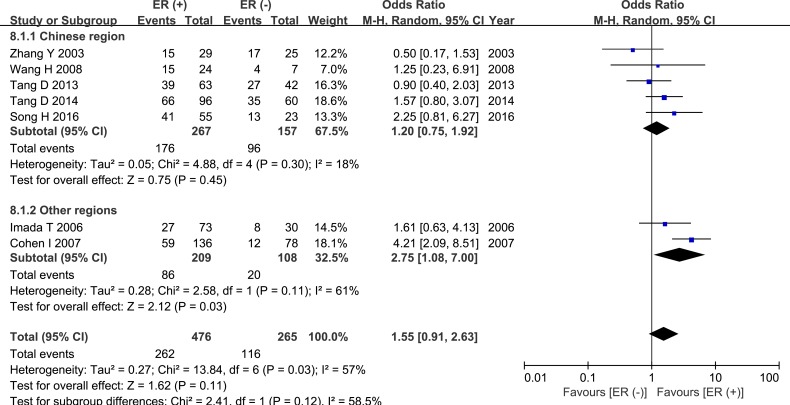
Meta-analysis of ER status and HPSE expression

**Figure 9:**
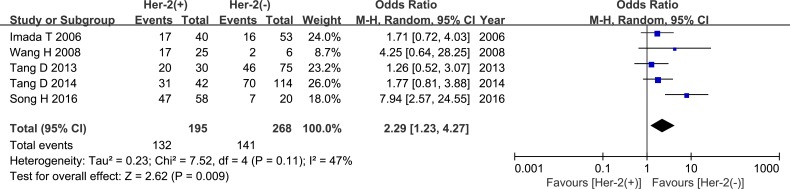
Meta-analysis of HER-2 status and HPSE expression

Seven studies compared HPSE expression in ER-positive and ER-negative breast cancer groups. These studies included 741 cases; 476 were in the ER (+) group and 265 cases were in the ER (−) group. Meta-analysis revealed a trend towards higher HPSE expression in the ER (+) group than in the ER (−) group, but this difference did not reach statistical significance (OR = 1.55, 95% CI = 0.91 – 2.63, *P* = 0.11, Figure 8, Table 2). In addition, there was significant inter-study heterogeneity (*P* = 0.03), and subgroup analysis revealed that the regional distribution of the research sites was the source of the heterogeneity. High HPSE expression was also correlated with HER-2 status (OR = 2.29, 95% CI = 1.23 – 4.27, *P* = 0.009, Figure 9, Table 2).

### Elevated heparanase expression is associated with poor 5-year survival

**Figure 10:**
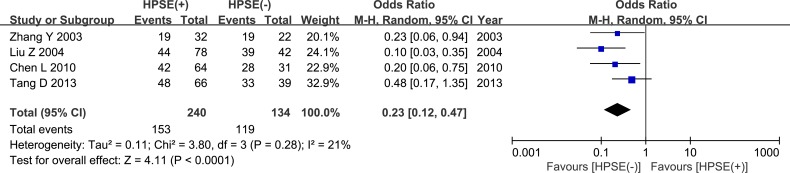
Meta-analysis of 5-year survival

The pooled analysis from 4 studies showed that HPSE expression was associated with poor 5-year survival (OR = 0.23, 95% CI = 0.12 – 0.47, *P* < 0.0001, Figure 10, Table 2). This analysis included a total of 374 patients, with 240 patients in the HPSE-positive group and 134 patients in the HPSE-negative (control) group. There was no significant inter-study heterogeneity *(P* = 0.28). Another study conducted by Davidson *et al.* found only a trend towards worse overall survival (OS) in patients with effusions containing HPSE-expressing tumor cells; however, because a different type of data was used in that study, it was not included in our meta-analysis.

### Publication bias

**Figure 11:**
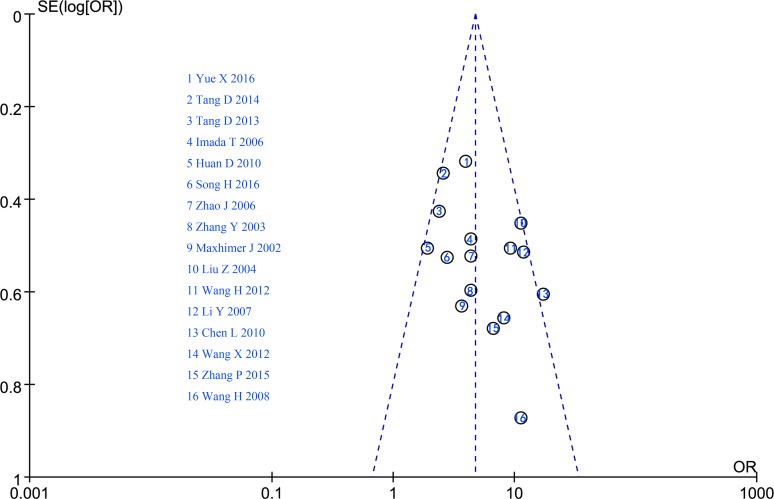
Funnel plot

A funnel plot was used to evaluate potential publication bias based on LNM; the relatively symmetrical funnel plot revealed no significant bias among the studies (Figure 11).

### HPSE and breast cancer: an analysis using TCGA data

**Figure 12:**
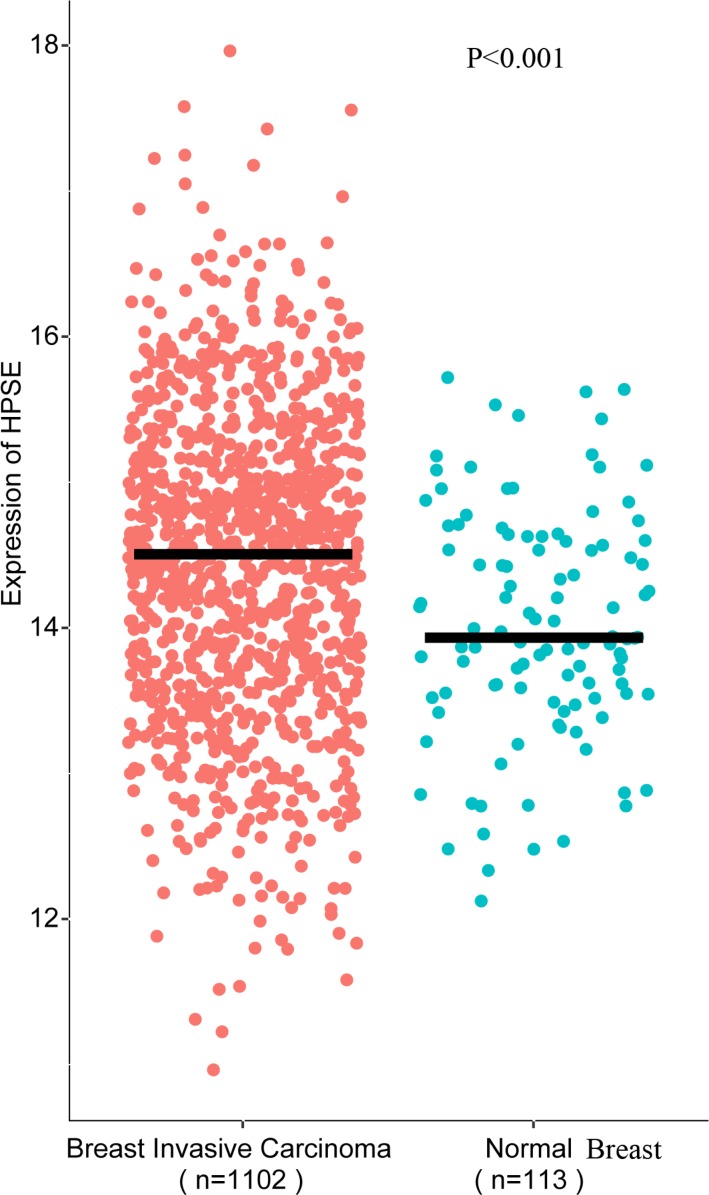
HPSE was highly expressed in breast invasive carcinoma compared to the normal breast tissue

**Figure 13:**
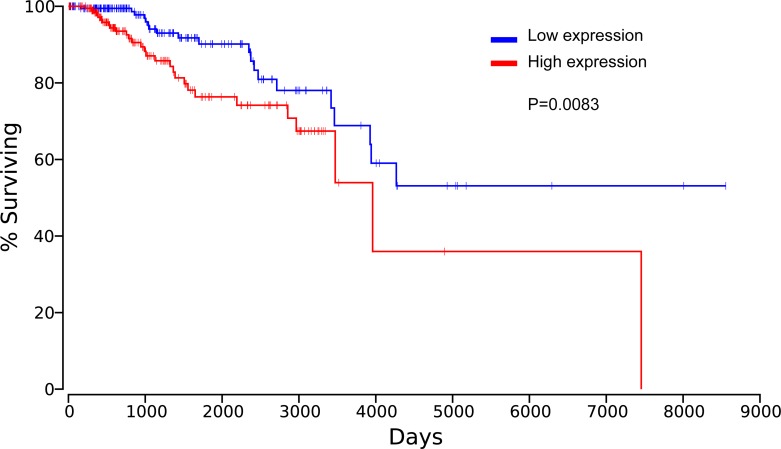
Elevated HPSE expression was associated with reduced OS in breast invasive carcinoma

The association of HPSE and breast cancer was also evaluated from TCGA data. The result suggested that HPSE was highly expressed in breast invasive carcinoma (BRCA) compared to the normal breast specimens (*P* < 0.001, Figure 12). Meanwhile, the analysis from TCGA data showed elevated HPSE expression was associated with reduced OS, significant difference between the top 20% HPSE expression group and the bottom 20% HPSE expression group was observed in the Kaplan plot generated by OncoLnc (*P* = 0.0083, Figure 13).

## DISCUSSION

HPSE promotes tumor cell invasion and metastasis specifically by degrading the extracellular matrix and increasing angiogenesis. This relationship between HPSE expression and tumor cell metastasis was first reported in 1983 [41]. Subsequent studies found that HPSE promotes capillary formation and increases microlymphatic vessel density. Possible molecular mechanisms underlying these effects include the following: (1) HPSE degrades HS, thereby abolishing its functions as an extracellular matrix barrier; (2) Cytokines that are bound by HS, such as FGF and VEGF, are released, thereby promoting tumor cell invasion and metastasis; and (3) Biologically active, HPSE-digested HS fragments create a microenvironment that promotes tumor cell survival [8, 42, 43].

The relationship between HPSE and breast cancer progression has been extensively examined in preclinical studies, but clinical evidence is limited. Here, we performed a study based on systematic review and TCGA data that used clinical samples to examine this relationship. We found that HPSE was overexpressed in breast cancer tissue compared to normal breast tissue. High HPSE expression was associated with increased LNM, larger tumors, higher histological grades, and poorer survival, suggesting that HPSE might be a promising prognostic marker. HPSE expression is elevated in highly metastatic tumor cells, and transfection of HPSE into non-metastatic tumor cells increased their metastatic ability (vs. control), while HPSE knockout decreased invasive and metastatic ability [44]. These results indicate that intrinsic HPSE activity plays an important role in tumor progression. Importantly, a recent study found that HPSE in the tumor microenvironment also promoted tumorigenesis [45], and inhibiting HPSE that originates from the tumor ECM can suppress tumorigenesis. Cross-talk between cancer cells and the ECM contributes to tumorigenesis, and HPSE modulates cancer progression by altering this cross-talk. In an unpublished study, we found that tumor growth and lung metastasis were increased in transgenic mice with high HPSE levels compared to the control group. These data are consistent with our current findings that increased HPSE expression is associated with more advanced clinical characteristics.

Although the difference did not reach statsitical significance, we also found that HPSE expression tended to be higher in ER-positive patients than in ER-negative group, which is consistent with results from previous studies [18, 19]. ER status is correlated with HPSE expression, and experiments have confirmed that HPSE acts downstream of ER signaling to promote breast cancer progression [18, 46]. *In vitro* experiments have demonstrated that tamoxifen induces high HPSE expression in breast cancer cells by increasing amplified in breast cancer 1 (AIB1) levels, which may partially explain the failure of tamoxifen treatments in ER-positive patients [18]. Furthermore, several studies have found that HPSE expression is elevated in treatment-resistant cancer cells. In addition, administration of an HPSE inhibitor attenuated chemo-resistance [20]. These results, together with the present meta-analysis, highlight the importance of HPSE as a predictive factor for breast cancer prognosis in the clinical setting.

HPSE expression is elevated and associated with clinical characteristics in several types of carcinomas in addition to breast cancer [47–52]. For example, a meta-analysis of 27 studies confirmed that HPSE expression was correlated with clinicopathological features in gastric cancer patients [53]. Similarly, in our 2016 meta-analysis of data from 16 studies conducted in Chinese patients, HPSE expression was predictive of prognosis [54]. This research, in which we used a more comprehensive search method and a wider variety of sources, confirms this association between HPSE and clinicopathological features and prognosis.

Some limitations should be considered when interpreting the results of this study. First, although we searched and retrieved studies from several databases, few of the included studies reported negative results, suggesting that our results may be influenced by publication bias. Second, in the included studies, the clinical data sample size was small, and differences in the methods used to determine HPSE expression (immunohistochemistry, RT-PCR analysis) may have increased heterogeneity in the meta-analysis, perhaps reducing the generalizability of our conclusions. Furthermore, the small number of studies that used PCR limited us to subgroup analysis only, and the antibodies used for IHC staining differed among studies, possibly confounding our comparisons. To address these issues, we plan to update this review when it becomes possible to evaluate the relationship between breast cancer progression and HPSE expression based on both protein and genomic data. Third, the studies included in this analysis generally do not describe the details of sample selection, which may lead to selection bias. Moreover, few studies reported baseline HPSE levels for the experimental and control groups, which may lead to inaccurate conclusions. And, because of the controlled-access and limited analytical techniques, we cannot make full use of the TCGA data, we will continue this study to perform meta-analysis and take advantage of TCGA data in our update work of this study.

HPSE has been studied extensively as an anti-cancer target [8, 45, 55, 56]. Recombinant HPSE and high-throughput drug screening technologies have made possible the development of several HPSE inhibitors, including neutralizing antibodies, polypeptides, small molecules, and modified HS. *In vitro* and *in vivo* experiments have shown that some of these compounds exert promising anti-tumor effects. For example, HPSE inhibitors dramatically inhibited cell invasion and reduced tumor growth in animal models [2, 8, 57]. These results suggest that therapies that target HPSE might improve cancer treatment. In addition, an understanding of the molecular mechanisms underlying the effects of HPSE will facilitate rapid advances of breast cancer treatment. Interestingly, several Chinese herbal medicines that have been used as components of breast cancer treatment in a number of countries have shown anti-HPSE activity [58, 59]. Such medicines may represent a promising starting point for the development of new HPSE-related drugs. Although more large-sample clinical data is needed to validate our findings, the results of this study suggest that the use of HPSE as a predictive factor for clinical prognosis and as a treatment target would benefit breast cancer patients.

## MATERIALS AND METHODS

### HPSE and breast cancer: a systematic review and meta-analysis

### Inclusion criteria

Cohort studies and case-control studies published in Chinese or English were included, without restrictions regarding the locations in which they were conducted.

Only studies that evaluated correlations between breast cancer and HPSE expression were included. No restrictions were made with respect to the source or type of specimens used for HPSE testing or the assay and methods used. The two outcomes considered were differences in HPSE expression between breast cancer tissue and normal tissue (or adjacent tissue) and the relationships between HPSE expression and (1) survival outcome, (2) LNM, (3) tumor size and histology grade, and (4) ER and HER-2 status. The criteria for exclusion from the meta-analysis were as follows: (1) studies that did not report original research and data; (2) no description of assays and methods; (3) duplicate reports of the same data obtained the same site; and (4) study data was not available.

### Search and retrieval strategy

Two researchers independently searched the following databases for articles published by July 28, 2016: PubMed, EMBASE via OvidSP, MEDLINE via OvidSP, Cochrane Library, Web of Science, and the Chinese databases CNKI, VIP, and WanFang. Keywords such as “breast cancer”, “heparanase”, and “heparan sulfate enzyme” were used to search for papers published in Chinese. All search strategies were validated in several tests. Given the outstanding contributions of Professor Isra Vlodavsky in this field, we also manually searched for publications from his group and retrieved additional relevant publications from the references in his papers. The PubMed, Web of Science, and MEDLINE via OvidSP search strategies are shown below:

**Figure:**
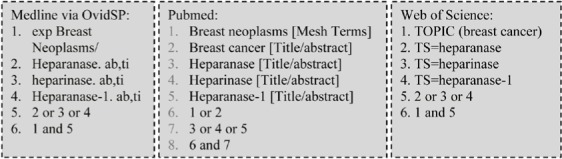


### Literature screening and data extraction

Endnote X7 was used as a literature management tool in this meta-analysis. Two researchers independently read the abstracts to exclude retrieved studies as needed based on the inclusion and exclusion criteria. The researchers also read the full text of articles that potentially met the requirements to determine whether they should be included. The results of this screening were then compared, and all of the researchers in the group met to make final decisions regarding any discrepancies. Two researchers independently used a pre-designed Excel spreadsheet to record the following extracted data: the name of the investigator; the year of publication; the source of samples; the target molecules analyzed; the assay methods; and the difference in HPSE expression and its correlation with clinical features of breast cancer.

### Quality assessment

Methodological quality was assessed using the Newcastle-Ottawa Scale (NOS). The scale includes 3 domains: selection, comparability, and outcome assessment. Studies with a scores of 7 to 9 were regarded as high quality. Two authors independently graded each study, and all of the researchers in the group met to make final decisions regarding any discrepancies.

### Statistical analysis

In the meta-analysis, RevMan 5.3 software was used for the meta-analysis of all data. The odds ratio (OR) statistic was used for non-continuous variables, and 95% confidence intervals (CI) were also determined. Due to variation in the HPSE assay reagents and procedures used in the selected studies, a random effects model was used for statistical analysis. In addition, a χ^2^ test was performed to analyze the heterogeneity of included studies that used similar measurements; *P* < 0.10 and I^2^ > 50% indicated significant heterogeneity. In the case of significant heterogeneity, subgroup analyses were conducted when possible to determine the source. A Z-test was performed to analyze total effects*; P* < 0.05 was considered statistically significant. A funnel plot was used to evaluate potential publication bias.

### HPSE and breast cancer: an analysis using TCGA data

As described, mRNA expression data of breast cancer were obtained from the Genomic Data Commons Data Portal (GDC) [60]. We downloaded the gene expression quantification file of breast invasive carcinoma and normal breast samples. Two-sided Wilcoxon rank-sum test was used to evaluate the gene expression difference of breast invasive carcinoma and normal breast tissue. Kaplan plot generated by OncoLnc (http://www.oncolnc.org) was performed to explore the association of HPSE and breast cancer. OncoLnc is a newly available resource for COX coefficients and linking TCGA survival data to mRNA, miRNA or lncRNA expression [61].
